# Role of the Gut Microbiome in Modulating Arthritis Progression in Mice

**DOI:** 10.1038/srep30594

**Published:** 2016-08-02

**Authors:** Xiaofei Liu, Benhua Zeng, Juan Zhang, Wenxia Li, Fangxiang Mou, Heng Wang, Qinghua Zou, Bing Zhong, Like Wu, Hong Wei, Yongfei Fang

**Affiliations:** 1 Department of Rheumatology, Southwest Hospital, Third Military Medical University, Chongqing, China; 2 Department of Laboratory Animal Science, College of Basic Medical Sciences, Third Military Medical University, Chongqing, China; 3 Department of Infectious Diseases, Chongqing Key Laboratory of Infectious Diseases, Southwest Hospital, Third Military Medical University, Chongqing, China

## Abstract

Genetics alone cannot explain most cases of rheumatoid arthritis (RA). Thus, investigating environmental factors such as the gut microbiota may provide new insights into the initiation and progression of RA. In this study, we performed 16S rRNA sequencing to characterise the gut microbiota of DBA1 mice that did or did not develop arthritis after induction with collagen. We found that divergence in the distribution of microbiota after induction was pronounced and significant. Mice susceptible to collagen-induced arthritis (CIA) showed enriched operational taxonomic units (OTUs) affiliated with the genus *Lactobacillus* as the dominant genus prior to arthritis onset. With disease development, the abundance of OTUs affiliated with the families Bacteroidaceae, Lachnospiraceae, and S24-7 increased significantly in CIA-susceptible mice. Notably, germ-free mice conventionalized with the microbiota from CIA-susceptible mice showed a higher frequency of arthritis induction than those conventionalized with the microbiota from CIA-resistant mice. Consistently, the concentration of the cytokine interleukin-17 in serum and the proportions of CD8+T cells and Th17 lymphocytes in the spleen were significantly higher in the former group, whereas the abundances of dendritic cells, B cells, and Treg cells in the spleen were significantly lower. Our results suggest that the gut microbiome influences arthritis susceptibility.

Rheumatoid arthritis (RA) is a common autoimmune disease that results in cartilage degradation, progressive disability, systemic complications, early death, and high socioeconomic cost. Genome-wide association studies and other surveys have identified several genetic susceptibility loci^1^, of which *HLA-DRB1* is the most predictive^2^. In addition, morbidity rates range from approximately 5% among dizygotic twins to 15–30% among monozygotic twins^3^. These observations indicate that the disease has a strong genetic component. Nevertheless, most cases of RA are of unknown cause, and environmental factors should be investigated.

One such environmental factor is the microbiota, which numerous studies have demonstrated to be a key element in the initiation and progression of many diseases, including metabolic disorders such as obesity, diabetes, and RA^4^. Moreover, metabolic productsfrom gut microbiota are believed to elicit autoimmune diseases in genetically susceptible individuals^5^. In particular, molecular mimicry by the microbiome may trigger RA^6^. Probiotic supplementation may, on the other hand, also relieve inflammation and other symptoms in arthritic patients^7^,^8^. Thus, we hypothesised that the composition, structure, and functional capacity of the gut microbiota are directly associated with the onset and progression of RA.

In DBA1 mice, arthritis can be stably induced in 80–100% of animals, using high-quality collagen^9^. This mouse model of arthritis shares immunological and pathological features with RA in humans and has been studied extensively to investigate pathogenesis and test candidate therapeutics. However, up to 20% of DBA1 mice do not develop arthritis symptoms after collagen-mediated induction. We speculated that microbial communities might also influence individual susceptibility or resistance to collagen-induced arthritis (CIA). To test this hypothesis, we analysed the microbiota in collagen-induced mice by surveying key bacterial phylotypes and other potential biomarkers before and after clinical onset of arthritis. Importantly, given that the gut microbiota shapes the development and activation of the mammalian immune system^10^, we showed that the transplantation of microbiota from CIA-susceptible mice into germ-free mice increased arthritis severity, the serum concentration of interleukin (IL)-17, and the proportions of CD8+ and IL-17-producing (Th17) T cells in the spleen.

## Results

### Mice with CIA have altered microbial diversity and composition

High-throughput sequencing of faecal samples generated 755,590 non-chimeric high-quality sequences and 704,057 reads. On average, 23,469 ± 6,794 quality reads were obtained per sample. Overall, 610 operational taxonomic units (OTUs) were detected with 3% dissimilarity between reads.

### Microbial richness decreased and diversity increased in mice with CIA

**Figure 1:**
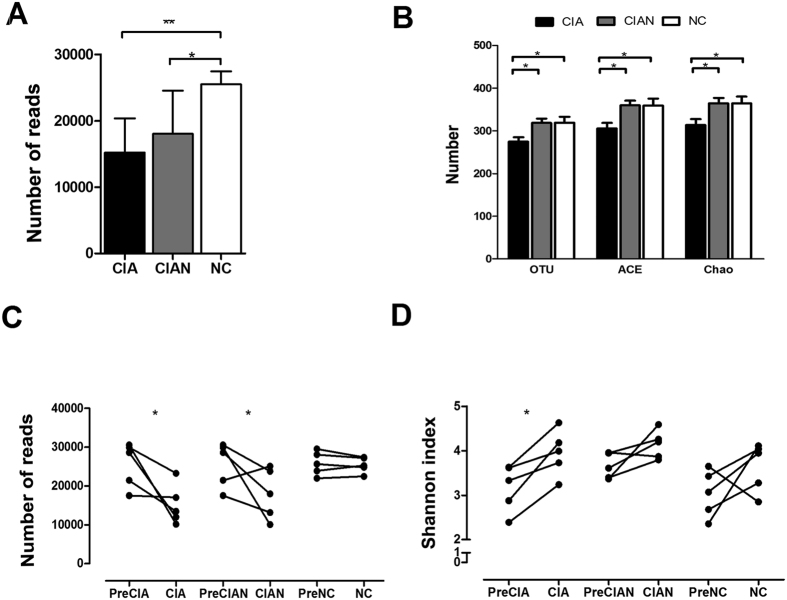
Analysis of microbial diversity. Mice were induced with collagen II on days 0 and 21. On day 35, mice that developed arthritis were classified as CIA-susceptible, and those that did not were classified as CIA-resistant. Faecal samples were collected before (on day 28) and after (on day 35) clinical onset of arthritis. (**A**) Number of reads for operational taxonomic units (OTUs) and (**B**) the ACE and Chao richness estimators per group were determined on day 35 (cross-sectional study). (**C**) Changes in the number of reads and (**D**) Shannon diversity index scores per group before and after collagen-mediated induction (cohort study). Each symbol represents an individual mouse. Error bars indicate SEM. Data in (**A**,**B**) were compared by one-way analysis of variance, while those in (**C**,**D**) were compared by paired *t*-test. *n* = 5; *p < 0.05; **p < 0.01. CIA, mice with collagen-induced arthritis; CIAN, mice without arthritis after collagen-mediated induction; NC, untreated control.

Reads, sequences, OTUs, ACE and Chao richness estimators, and Shannon and Simpson diversity indices were determined for untreated mice and for mice that were either CIA-susceptible (i.e. developed arthritis) or CIA-resistant (i.e. did not develop arthritis). Untreated mice had significantly richer reads than collagen-induced mice with or without arthritis (Fig. 1A). However, arthritic mice had fewer OTUs and lower ACE and Chao richness estimators than untreated mice or collagen-induced mice without arthritis (Fig. 1B). Indeed, sequence reads diminished considerably after collagen-mediated induction, irrespective of arthritis onset (Fig. 1C). Interestingly, comparing the changes of the diversity indices among groups revealed that the Shannon index increased significantly only in mice that developed arthritis after collagen-mediated induction (Fig. 1D).

### Bacterial compositiondiffered between mice susceptible and resistant to CIA

We next sought to identify bacterial phyla, families, and genera that could account for the differences in microbial richness and diversity. Notably, of the 15 phyla detected after collagen-mediated induction, seven were not detected after disease onset (Supplementary Figure 1). CIA-susceptible mice hosted the largest number of phyla before disease onset, of which the most abundant were Firmicutes (47.58%), Bacteroidetes (29.48%), Proteobacteria (14.98%), and Candidate division TM7 (6.14%). Deinococcus–Thermus and Verrucomicrobia were also detected in these mice after induction but were absent from CIA-resistant mice.

**Figure 2:**
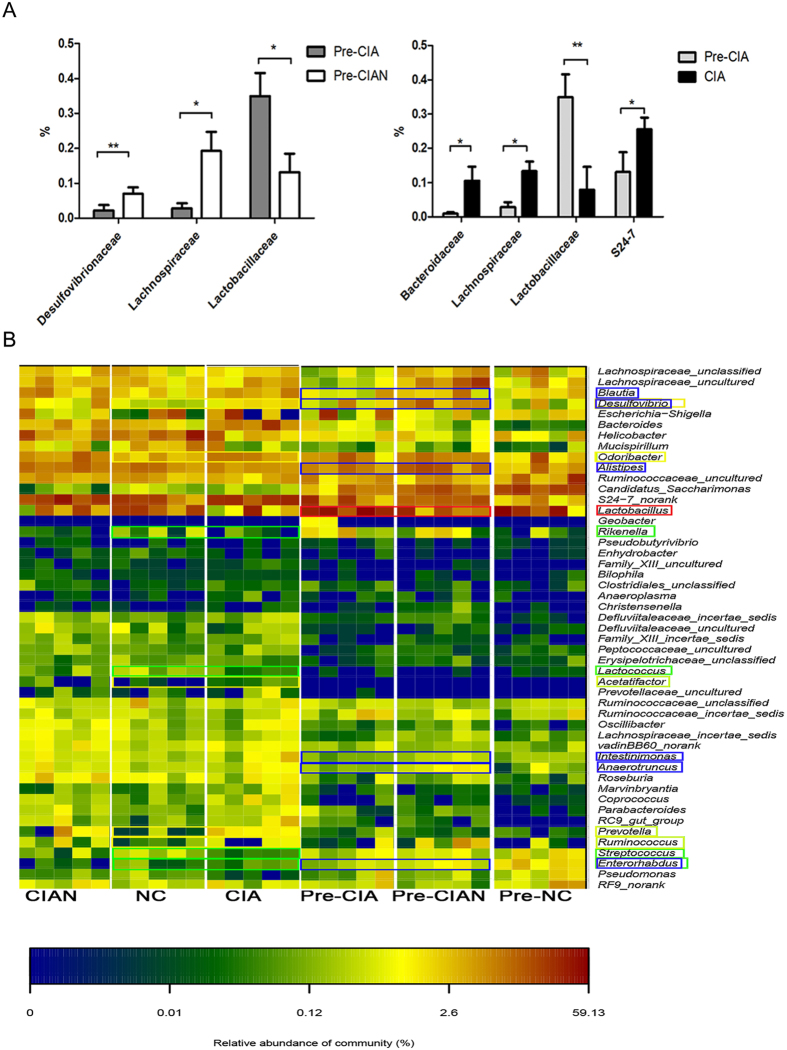
Differences in microbiome composition between CIA-susceptible and -resistant mice. (**A**) The distribution of bacterial families with relative abundance >1% in at least onesample was compared by Student’s *t*-test in mice prior to arthritis onset (left panel) and by Student’s paired *t*-test in CIA-susceptible mice (right panel). *n* = 5 per group; *p < 0.05; **p < 0.01. (**B**) Heat map of the relative abundances of genera in each group. The vertical columns represent one group (*n* = 5 mice per group), and the horizontal rows depict genera. The colour coding ranges from blue (not detected), through green (low abundance) and yellow (medium abundance), to red (high abundance). The genera shown with red and blue blocks were more abundant in CIA-susceptible mice prior to arthritis onset and CIA-resistant mice, respectively. Yellow and green blocks indicate genera with greater abundance in CIA and untreated control (NC) mice, respectively.

We also found that the bacterial families Desulfovibrionaceae (*p* < 0.01) and Lachnospiraceae (*p* < 0.05) were less abundant in CIA-susceptible mice than in CIA-resistant mice before disease onset (Fig. 2A, left panel). Notably, the family Lactobacillaceae was significantly more abundant in CIA-susceptible mice (*p* < 0.05), and the genus *Lactobacillus* was significantly more abundant after collagen-mediated induction in CIA-susceptible mice than in CIA-resistant mice (*p* < 0.001, Supplementary Table 1). We also found that the families Bacteroidaceae, Lachnospiraceae, and S24-7 increased significantly after arthritis onset (Fig. 2A, right panel).

At the genus level, genera significantly less abundant in CIA-susceptible mice prior to arthritis onset than in CIA-resistant mice included *Enterorhabdus*, *Alistipes*, *Desulfovibrio*, and others (Fig. 2B and Supplementary Table 1). After arthritis onset, *Enterorhabdus*dramatically increased in abundance as arthritis progressed in CIA-susceptible mice. In contrast, *Pseudomonas* spp. was significantly less abundant in CIA-susceptible mice (Supplementary Table 2). When compared with their corresponding proportions in the microbiota of untreated mice, *Enterorhabdus, Myroides*, *Rikenella*, *Brochothrix*, *Lactococcus*, and *Streptococcus* were less abundant in arthritic mice, whereas *Desulfovibrio*, *Prevotella*, *Parabacteroides*, *Odoribacter*, *Acetatifactor*, *Blautia*, *Coprococcus*, and *Ruminococcus* were more abundant in arthritic mice (Supplementary Table 2).

### Collagen-mediated arthritis induction and CIA-susceptibility separate mice based on β-diversity

**Figure 3:**
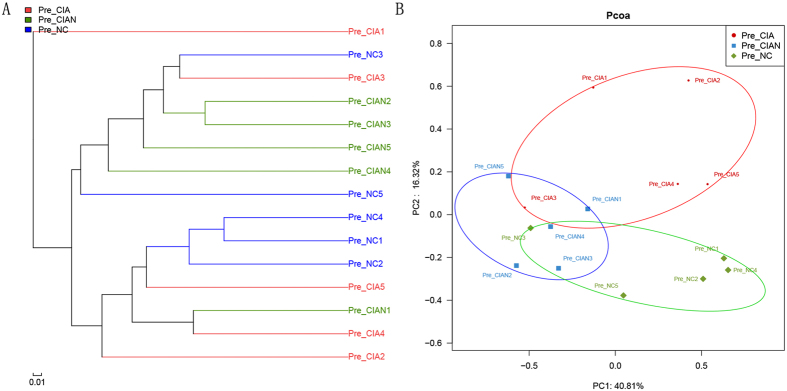
Separation of mice according to their susceptibility to CIA. (**A**) Clustering of unweighted UniFrac distances between samples colour-coded by breeding pair, as determined by the UniFrac-based unweighted pair group method with arithmetic analyses. (**B**) The top two principal components of unweighted UniFrac distances between untreated and collagen-induced mice prior to arthritis onset. Symbols represent data from individual mice. Red circles indicate CIA-susceptible mice. Blue and green circles indicate CIA-resistant and untreated mice, respectively.

Fast UniFrac and Bray–Curtis dissimilarity analyses confirmed that the observed trends were not specific to the analytical methods applied. In particular, the UniFrac-based unweighted pair group method with arithmetic analyses showed that the composition of the bacterial community differed between CIA-susceptible and -resistant mice after collagen-mediated induction (Fig. 3A). Principal component analysis of OTUs scored by Bray–Curtis dissimilarity also revealed that CIA susceptibility and collagen-mediated induction shaped the microbial community, with a clear separation of samples according to these conditions prior to arthritis onset (Fig. 3B). Divergence in the distribution of the microbiota prior to disease onset was pronounced and statistically significant (ANOSIM, R = 0.375, *p* < 0.01). Similar visual separation was achieved by nonmetric multidimensional scaling (NMDS) ordination of Bray–Curtis dissimilarity metrics (Supplementary Figure 2). Furthermore, the results indicated significant differences in bacterial diversity among groups (ANOSIM, R = 0.605, *p* < 0.001).

### Germ-free mice conventionalized with microbiota from CIA-susceptible or -resistant mice show microbiome differences

**Figure 4:**
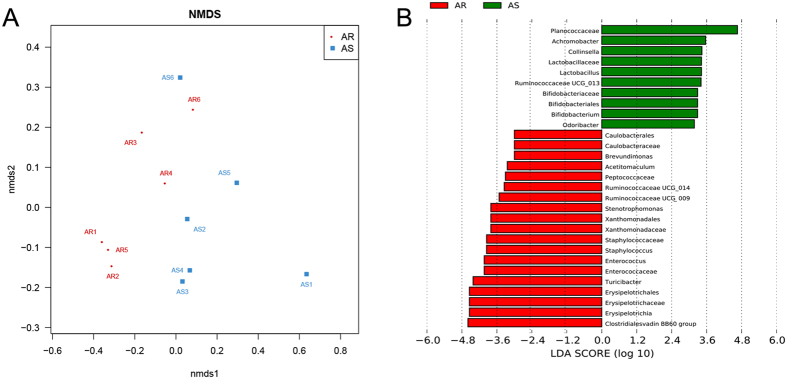
Analyses of faecal microbiomes of germ-free mice conventionalized with CIA-susceptible or CIA-resistant mice. (**A**) NMDS ordination for all 12 mice following conventionalization with the microbiome of CIA-susceptible or CIA-resistant mice. (**B**) LEfSe-identified LDA bar graphs of taxa/clades with differential abundance following conventionalization with the microbiome of the CIA-susceptible (green) and the CIA-resistant (red) mice. Taxa in this graph were statistically significant (*p* < 0.05) and had an LDA Score > ± 2.0, which was considered a significant effect size.

To determine whether the microbiome contributes directly to arthritis progression, we conventionalized germ-free mice (*n* = 6) with the microbiome of either CIA-susceptible or CIA-resistant mice. To ensure that mice were colonized reproducibly and stably, we transplanted faecal samples to two independent groups and analysed the microbial community structures in each group of six mice. Using community-wide β-diversity analyses, we determined that conventionalization with these two treatments resulted in two distinct microbial community structures (ANOSIM, *p *= 0.019) (Fig. 4A).

While NMDS ordination can summarize multidimensional clustering, it cannot identify specific bacterial taxa that are differentially represented in our two groups. We therefore used the LDA Effect Size (LEfSe) algorithm to identify the specific clades that are differentially represented in cases versus controls. By specifying control and case status as distinct classes, we found that, similar to CIA-susceptible SPF mice, germ-free mice conventionalized with the microbiome of susceptible mice showed enrichment in the relative abundance of OTUs affiliated with the genus*Lactobacillus* compared to germ-free mice conventionalized with the microbiome of resistant mice (Fig. 4B).

### Microbiota from CIA-susceptible mice increased arthritis incidence and severity in germ-free mice

**Figure 5:**
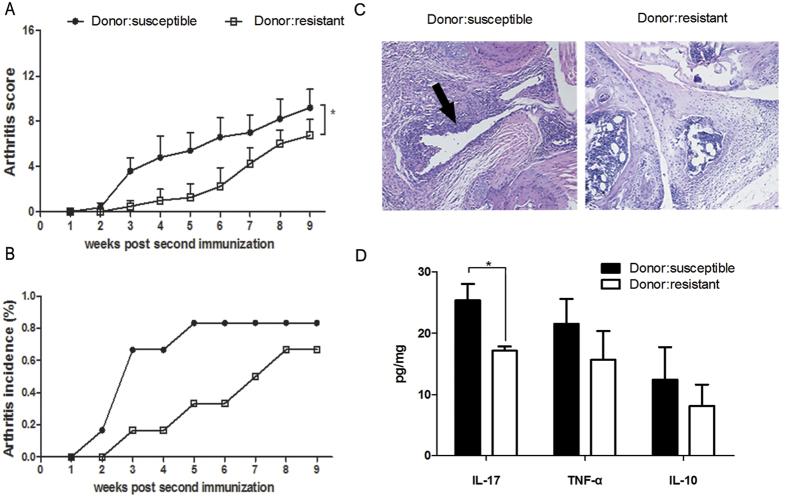
Higher incidence and severity of arthritis in CIA-susceptible mice. Germ-free mice were conventionalized with microbiota obtained from CIA-susceptible or CIA-resistant mice after induction with collagen. Four weeks after conventionalization, recipient mice were induced with collagen II under germ-free conditions. (**A**) Clinical scores progression was assessed, and (**B**) the incidence of arthritis was determined. Data indicate means ± SEM from six animals per group. *p < 0.05. (**C**) Representative sections of ankle joint tissue stained with hematoxylin and eosin. Magnification, 100×. Histopathological evaluation revealed severe inflammation in the joint sections of CIA mice (arrowhead). (**D**) Serum tumour necrosis factor-α, interleukin-17, and interleukin-10 as estimated by enzyme-linked immunosorbent assay. Data indicate mean ± SEM (*n* = 6). *p < 0.05 by Student’s *t-*test.

Following conventionalization, mice were induced with collagen II under germ-free conditions as previously described. Mice conventionalized with the microbiome from CIA-susceptible mice showed increased arthritis scores (Fig. 5A) and arthritis incidence (Fig. 5B) relative to those conventionalized with the microbiota from CIA-resistant mice. Arthritic joints were examined by histopathology (Fig. 5C), and the arthritic changes were also more severe in the former group.

In addition, we measured serum levels of pro- and anti-inflammatory cytokines. The concentrations of IL-17 and tumour necrosis factor (TNF)-α were positively correlated with joint damage, whereas that of IL-10 was negatively correlated (Fig. 5D). We found that the serum concentration of IL-17 was consistently elevated in germ-free mice colonized with the microbiota from CIA-susceptible mice (Fig. 5D). The concentrations of TNF-α and IL-10 also increased in the same group of mice, but the change was not statistically significant.

### Microbiota from CIA-susceptible mice increased Th17 and reduced T regulatory (Treg) cell proportions

**Figure 6:**
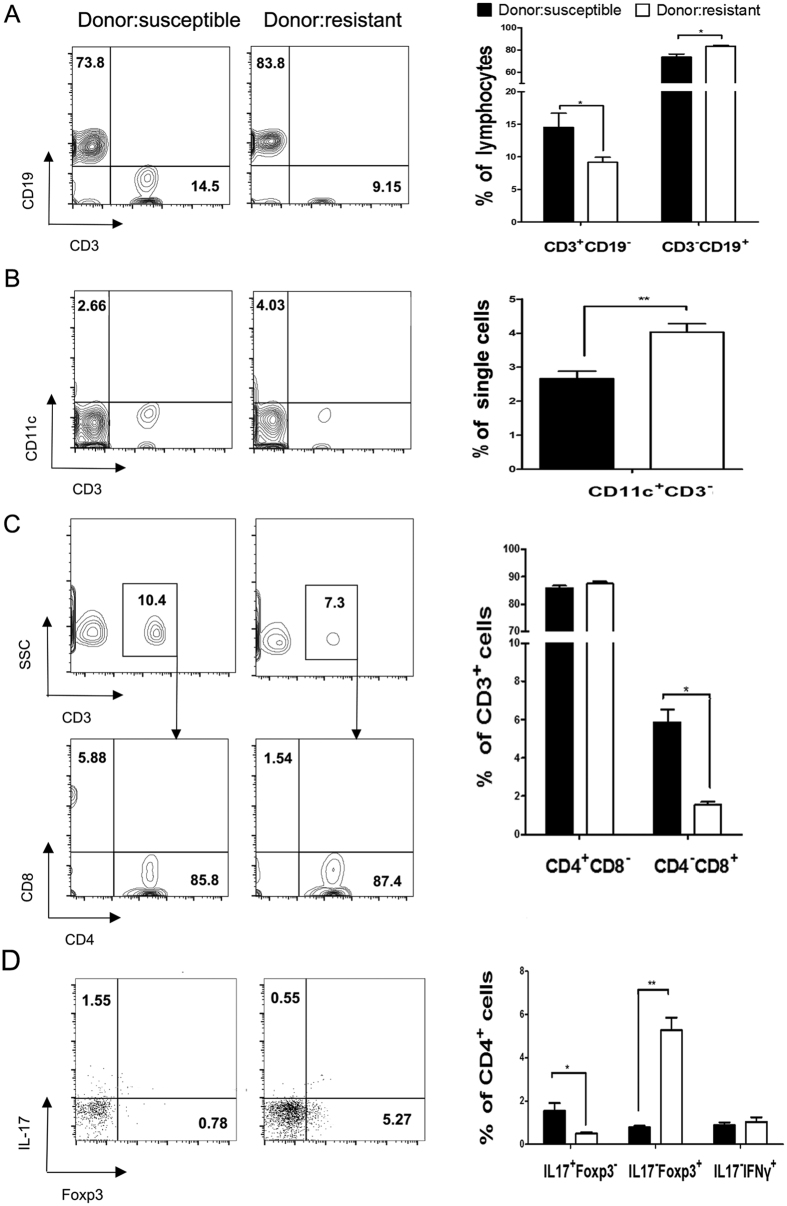
Phenotypic analysis of splenocytes from germ-free mice conventionalized with the microbiota from CIA-susceptible or -resistant mice. Lymphocytes were harvested from the spleen after 9 weeks of immunization with collagen. Cells were stained with CD3, CD19, CD11c, CD4, and CD8 antibodies and analysed by flow cytometry. Representative contour plots showing CD3 vs. CD19 (**A**, left panel) and CD11c (**B**, left panel), and CD4 vs. CD8 (**C**, left panel) expression. Data indicate mean ± SEM (*n* = 6). *p < 0.05; **p < 0.001 by Student’s *t-*test. CD3-CD19+ B cells (**A**, right panel) and CD3-CD11c+ dendritic cells (**B**, right panel) were more abundant in germ-free mice conventionalized with the microbiota from CIA-resistant mice than in those conventionalized with the microbiota from CIA-susceptible mice. CD4-CD8+ T cells (**C**, right panel) were more abundant in recipients of CIA-susceptible microbiota. CD4+ T cells were stained with intracellular anti-IFN-γ antibody, anti-Foxp3 antibody, and anti-IL-17A antibody. Numbers in each quadrant indicate the percentages of CD4+ T cells positive for each cytokine (**D**, right panel). Mean ± SEM of the IL17+Foxp3-, IL17-Foxp3+, and IL17-IFN-γ+ T cell subsets (**D**, left panel). Data indicate mean ± SEM (*n* = 6). *p < 0.05; **p < 0.001 by Student’s *t-*test.

We measured the abundances of T cells, CD4+ T cells, CD8+ T cells, and professional antigen-presenting cells (APCs) including B cells and dendritic cells (DCs). CD4+ T cells are required in the aetiology of CIA, and CD8+ T cells can clear cells infected with pathogenic organisms^11^,^12^. APCs are involved in the maintenance of tolerance to the commensal microbiota and the generation of protective immune responses against pathogens. Conventionalization of germ-free mice with the microbiota from CIA-susceptible mice significantly decreasedthe abundances of CD3-CD19+B cells (Fig. 6A) and CD3-CD11c+ DCs (Fig. 6B). However, the abundances of total and CD8+ T cells (Fig. 6C) significantly increased. Interferon (IFN)-γ producing (Th1), Th17, and Treg lymphocytes are key players in RA^13^. Conventionalization with the microbiota from CIA-susceptible mice significantly increased the abundance of Th17 cells (*p* < 0.05) and reduced that of Treg cells (*p* < 0.01) (Fig. 6D).

## Discussion

The gut microbiota is considerably and reproducibly influenced by dietary factors, as well as by lifestyle, ethnicity, and geographic origin^14^. Therefore, arthritic and germ-free animal models are more suitable for investigating the relationship between disease and the host microbiome. We hypothesised that the gut microbiome is associated with CIA in mice.

As has been noted, not all mice induced with collagen developed arthritis, indicating that collagen is not sufficient to trigger the disease. Rather, our results indicate that the combined effects of collagen-mediated induction and changes in the microbiome as a result of such induction might be necessary to induce arthritis. Indeed, the data suggest that collagen-mediated induction altered the microbial diversity in CIA-susceptible mice, with a significantly increased Shannon index and reduced species richness in these mice, but not in CIA-resistant mice.

Differences in the composition of the gut microbiota were consistently detected between arthritis-susceptible and healthy or arthritis-resistant hosts. We found that the genus *Lactobacillus*was significantly more abundant in CIA-susceptible mice prior to arthritis onset than in CIA-resistant mice. This observation agrees with our previous studies, in which we found that *Lactobacillus* strains including *L. salivarius, L. iners,* and *L. ruminis* were more abundant in patients with RA than in healthy controls^15^. Similarly, recent surveys demonstrated that *L. salivarius* was over-represented in the faecal, dental, and salivary microbiota of patients with RA, and its abundance was particularly pronounced in severe cases^16^. These findings indicate that *L. salivarius* might be a marker of RA. In addition, some *Lactobacillus* species might cause arthritis^17^. In at least one study, *L. bifidus* could induce joint swelling in germ-free mice^18^. It has been shown that commensal strains of the genera *Lactobacillus* and *Bifidobacterium* can improve the effector function of CD8+ T cells in the intestinal mucosa^19^. In the present study, we observed that germ-free mice that were conventionalized with the microbiota of CIA-susceptible mice, which provided them with an increased proportion of *Lactobacillus* spp. and *Bifidobacterium* among other changes, had an increased abundance of CD8+ T cells in spleen.

The prevalences of the family Lachnospiraceae and genus *Enterorhabdus* were lower in CIA-susceptible mice prior to arthritis onset than in CIA-resistant mice, but significantly increased after disease onset in CIA-susceptible mice. Bacteroidaceae, Lachnospiraceae, and S24-7 increased significantly in CIA-susceptible mice following the onset of CIA, which contributed to the observed increase in taxonomic diversity during the progression of CIA. S24-7 and Bacteriodales protect against disease whereas members of Lachnospiraceae promote pathogenesis^20^. Furthermore, Lachnospiraceae bacterium A4 can promote Th1 and Th17 polarization and down-regulate Th2 responses^21^. In our study, we observed that germ-free mice conventionalized with the microbiota from CIA-susceptible mice had more Th17 cells in the spleen. Notably, the abundances of the genera *Desulfovibrio and Alistipes* were lower in CIA-susceptible mice prior to arthritis onset than in CIA-resistant mice*. Desulfovibrio* and *Alistipes* have been associated with mucosal thickness^22^. Moreover, *Pseudomonas* spp. was significantly less abundant in CIA-susceptible mice after disease onset than in CIA-resistant mice, and it was previously reported that the administration of the *Pseudomonas* exotoxin A translocation domain significantly impeded progression of arthritis in mice^23^.

When compared with their corresponding proportions in the microbiota of untreated mice, *Prevotella* species were more abundant in arthritic mice. *Prevotella*was extensively characterised in a previous study^24^. Notably, we did not observe changes in the prevalence of *Porphyromonas gingivalis*, the major causative agent of periodontitis that also facilitates the development and progression of CIA^25^.

Due to the extreme complexity and diversity of the gut microbiome, it is unlikely that differences in the abundance of a single species are responsible for differences in RA susceptibility. Rather, these differences most likely result from context-dependent interactions among the multiple species constituting the gut microbiota. Furthermore, resident bacteria have profound effects on proximal and systemic inflammation and immunity^26^,^27^ and might therefore play a role in the development of arthritis.

Consistent with this notion, divergence in the composition of the microbiota composition between CIA-susceptible and CIA-resistant mice before the onset of CIA was pronounced and statistically significant. Therefore, we conducted further experiments to determine the effect of transferring the microbiota from the CIA-susceptible and CIA-resistant mice to germ-free mice by faecal transplantation. We found that germ-free mice conventionalized with microbiota from CIA-susceptible mice developed arthritis at a higher frequency and with higher severity than those transplanted with CIA-resistant microbiota. In addition, we observed significant differences in the abundances of selected types of splenic lymphocytes and in the concentrations of certain inflammatory cytokines in serum.

The pathology of RA involves inappropriate inflammatory responses mediated by CD4+, Th1, Th17, and Treg lymphocytes. In our study, the proportion of Th17 cells in the spleen was higher and that of Treg cells was lower in mice conventionalized with the microbiota from CIA-susceptible mice than in those conventionalized with the microbiota from CIA-resistant mice. The concentration of IL-17 in serum was also higher in the former group, whereas the proportion of Th1 lymphocytes in the spleen did not differ significantly. Th17 cells and IL-17 are considered key drivers of joint, cartilage, and bone damage^28^. Commensal bacteria influence T helper cell differentiation, immune responsiveness, and homeostasis^29^. There is strong evidence suggesting that segmented filamentous bacteria^30^,^31^ and bacteria in the family Lachnospiraceae promote Th17 cell polarization^21^, whereas *Bacteroides fragilis*^32^, *Clostridium* spp.^33^, and *Bifidobacterium infantis*^34^ induce Treg cells. The abundance of the family Lachnospiraceae increased significantly after the development of CIA in our study. These findings support the hypothesis that arthritis induction by collagen in DBA1 mice is associated with the composition of the gut microbiome.

The differentiation of T cells is tightly regulated by cytokines derived from APCs, including DCs and B cells^35^. Intestinal colonization with a specific commensal bacterium can provide protection via extra-intestinal effects on DCs^36^. Under certain conditions, immature DCs can become tolerogenic and promote differentiation of Tregs^37^. DCs affect IgA production from B cells and influence the generation of unique subsets of T cells^38^. We found that germ-free mice that were conventionalized with the microbiota from CIA-resistant mice showed increased abundances of CD11c+DCs and CD19+B cells. Transferring microbiota to antibiotic-treated or germ-free mice can increase granulocyte-macrophage colony-stimulating factor (GM-CSF) levels^39^. GM-CSF is generally recognized as an inflammatory cytokine for its role as a growth and differentiation factor in granulocyte and macrophage populations; it functions as an anti-inflammatory/regulatory cytokine in certain types of autoimmune diseases such as RA, multiple sclerosis, Crohn’s disease, and type-1 diabetes^40^,^41^,^42^. GM-CSF modulates differentiation of DCs to render them ‘tolerogenic’, which can increase the numbers of circulating Foxp3+ cells and Foxp3 expression levels in Tregs^43^,^44^.

There are some new therapeutic strategies for RA that involve the induction of specific tolerances such as eye-induced tolerance^45^, which can induce specific immune tolerance via CD8+Tregs^46^, or oral tolerance 47. For example, previous oral administration of type II collagen (CII)can inhibit the development of CIA. DCs in the gut-associated lymphoid tissue take up the antigen and present it to T cells to generate Tregs, which induce systemic immune tolerance to CII^48^; splenic IDO+CD11b+DCs also contribute to the systemic immune regulation in oral tolerance^49^. Commensal microbiota are known to play a critical role in the establishment of oral tolerances^50^,^51^. Studies in germ-free mice have established key roles for microbiota in the development, training, and function of the host immune system^52^. An understanding of the microbiota-associated mechanism would give clinicians new insights into the development of natural immune tolerance and new therapeutic approaches for the treatment of RA.

Taken together, our results demonstrated differences between the microbiome compositions of CIA-susceptible and CIA-resistant mice. When transplanted to germ-free mice, the microbiome of the former could aggravate disease severity in CIA, indicating a causal relationship between the composition of the gut microbiota and CIA susceptibility. These findings reveal that the intestinal microbiota profoundly affect the balance between pro- and anti-inflammatory immune responses during collagen-mediated induction. Further studies are necessary to explore the mechanisms underlying the protective or predisposing role of the gut microbiota in RA and leverage these to develop novel therapeutic approaches.

## Materials and Methods

### Statement of ethics of animal care and use

Animal protocols were approved by the Animal Care and Use Committee of the Southwest Hospital, Third Military Medical University, China, and were in accordance with “Principles for Use of Animals” and “Guide for the Care and Use of Laboratory Animals” of the U.S. National Institutes of Health.

### Experimental design and animals

Thirty DBA1 mice aged 8 weeks and weighing 18–20 g were obtained from the Shanghai SLAC Laboratory Animal Corporation (Production License No. 2007000573633) (Shanghai, China). Mice were housed under specific pathogen-free (SPF) conditions at the Department of Laboratory Animal Science, Third Military Medical University, Chongqing, China, with 44–65% humidity and 12-h light/dark cycle, at 22–26 °C. Mice were then bred in-house for at least three generations and provided with a sterile diet and water to ensure adaptation to housing conditions and to obtain germ-free mice.

### Induction and assessment of arthritis

DBA1 mice were induced with a subcutaneous injection of 2 mg/mL bovine type II collagen (Chondrex, Redmond, WA, USA) emulsified in complete Freund’s adjuvant (Chondrex). On day 21, mice received a subcutaneous booster dose of 100 μL of collagen II without complete Freund’s adjuvant. Arthritis induction and severity were assessed by two investigators blinded to the treatment groups. Untreated mice were used as a control.

### Microbiota sampling, DNA extraction, and PCR

The gut microbiota in untreated (*n* = 5), CIA-susceptible (*n* = 5), and CIA-resistant mice (*n* = 5) were characterised before and after the clinical onset of arthritis under SPF conditions. Briefly, fresh faecal matter was collected from each mouse and immediately stored until analysis or transplantation into germ-free mice^53^. Total genomic DNA was isolated usinga QIAamp^®^ DNA Stool Mini Kit (Qiagen, Hilden, Germany) according to the manufacturer’s instructions. The DNA concentration was measured using a NanoDrop^®^ 1000 spectrophotometer (Thermo Scientific, Wilmington, DE, USA). The universal primers 338F (5′-ACTCCTACGGGAGGCAGCA-3′) and 806R (5′-GGACTACHVGGGTWTCTAAT-3′), which target V3–V4 of 16S rRNA, were used for amplification and subsequent pyrosequencing of PCR products. The amplification program consisted of initial denaturation at 95 °C for 2 min followed by 25 cycles of 95 °C for 30 s, 55 °C for 30 s, and 72 °C for 45 s. Amplicons were purified and sequenced using a MiSeq sequencer (Illumina, San Diego, CA, USA).

### Taxonomy assignment and community structure

Sequencing reads were processed using the Quantitative Insights into Microbial Ecology software program, and taxonomy was assigned using a naive Bayes classifier^54^. OTUs were annotated using the Ribosomal Database Project Naive Bayes classifier^55^. Principle rarefaction curves were generated in MOTHUR version 1.30.1, along with principal components, Chao, ACE, Shannon, and Simpson indices^56^. Taxonomic classification to phylum, class, order, family, and genus were also performed in MOTHUR using the SILVA database with confidence threshold 80%. R was used to generate heat maps and perform cluster analysis. Differential abundance was analysed in MetaStats^57^, and OTU-based clustering with genetic distance 0.03 was analysedby nonmetric multidimensional scaling.

### Gnotobiotic mouse experiments

Germ-free DBA1 mice were housed and maintained as previously described^58^,^59^ and inoculated by oral gavage with 0.2 mL mixed faecal suspension obtained from CIA-susceptible or CIA-resistant mice prior to collagen-mediated induction. All 24 mice were acclimated to laboratory conditions for 4 weeks, at which point 12 mice were induced with collagen II. Mice were housed in two independent isolators within four cages, with six mice per cage.

### Flow cytometry

Single-cell suspensions were isolated from the spleen and analysed using an Accuri C6™ flow cytometer (BD Biosciences, San Jose, CA, USA) using the following antibodies: anti-CD3 and anti-IL-17A conjugated to phycoerythrin (PE), anti-CD4 and anti-CD19 conjugated to allophycocyanin, anti-CD8a conjugated to PE/Cy5, anti-CD11c conjugated to PE/Cy7, anti-IFN-γ conjugated to fluorescein isothiocyanate (BioLegend, San Diego, CA, USA), and mouse Tregphenotyping cocktail (BD Biosciences). The data file was analysed as follows: exclusion of non-single events; gating of T cells (CD3+CD19−), B cells (CD19+CD3−), and DCs (CD11c+CD3−); gating of CD3+ T cells (anti-CD3 versus sideward scatter); further separation of CD3+T cells into CD4+ or CD8+ T cells (anti-CD4 versus anti-CD8); and further gating of CD4+T cells into TH17(IL17+Foxp3−), Treg (IL17-Foxp3+), and Th1 (IL17-IFN-γ+) subsets^60^,^61^,^62^.

### Cytokine assay

IL-10, TNF-α, and IL-17 were measured byenzyme-linked immunosorbent assays (Ebioscience, San Diego, CA, USA) following the manufacturer’s protocols.

### Histology

In brief, 5-μm sections were obtained from the right hind knee joint, stained with hematoxylinandeosin, and imaged at 100× magnification.

### Statistical analysis

The microbiomes of the same mouse before and after clinical onset of arthritis were compared by paired *t*-test. Differences in the numbers of reads were compared among groups using one-way analysis of variance. Differences in divergence in the distribution of microbiota were compared among groups using ANOSIM. Differences in arthritis incidence were compared between groups using Fisher’s exact test. Clinical scores were compared between groups using Wilcoxon signed ranks test. Serum cytokines and flow data were compared between groups using Student’s *t*-test. Data were analysed in SPSS 17 (IBM, Armonk, NY, USA), and *p*-values less than 0.05 were considered significant.

## Additional Information

Accession codes: Sequencing data have been deposited in GenBank Sequence Read Archive under accession number SRP058227. 

**How to cite this article**: Liu, X. *et al.* Role of the Gut Microbiome in Modulating Arthritis Progression in Mice. *Sci. Rep.* **6**, 30594; doi: 10.1038/srep30594 (2016).

## Supplementary Material

Supplementary Information
