# Supplementary material for: Role of the Gut Microbiome in Modulating Arthritis Progression in Mice
Source: Sci Rep. 2016 Aug 2;6:30594. doi: 10.1038/srep30594 (PMC4969881; doi:10.1038/srep30594)
Supplement: Supplementary Information [file srep30594-s1.pdf]

Supplementary information

Role of the Gut Microbiome in Modulating Arthritis Progression in Mice

Xiaofei Liu, Benhua Zeng, Juan Zhang, Wenxia Li, Fangxiang Mou, Heng Wang,Qinghua Zou, Bing Zhong, Like Wu, Hong Wei, &Yongfei Fang

Supplementary Figure 1. Bacterial species compositions of microbiota communities at the phylum level.

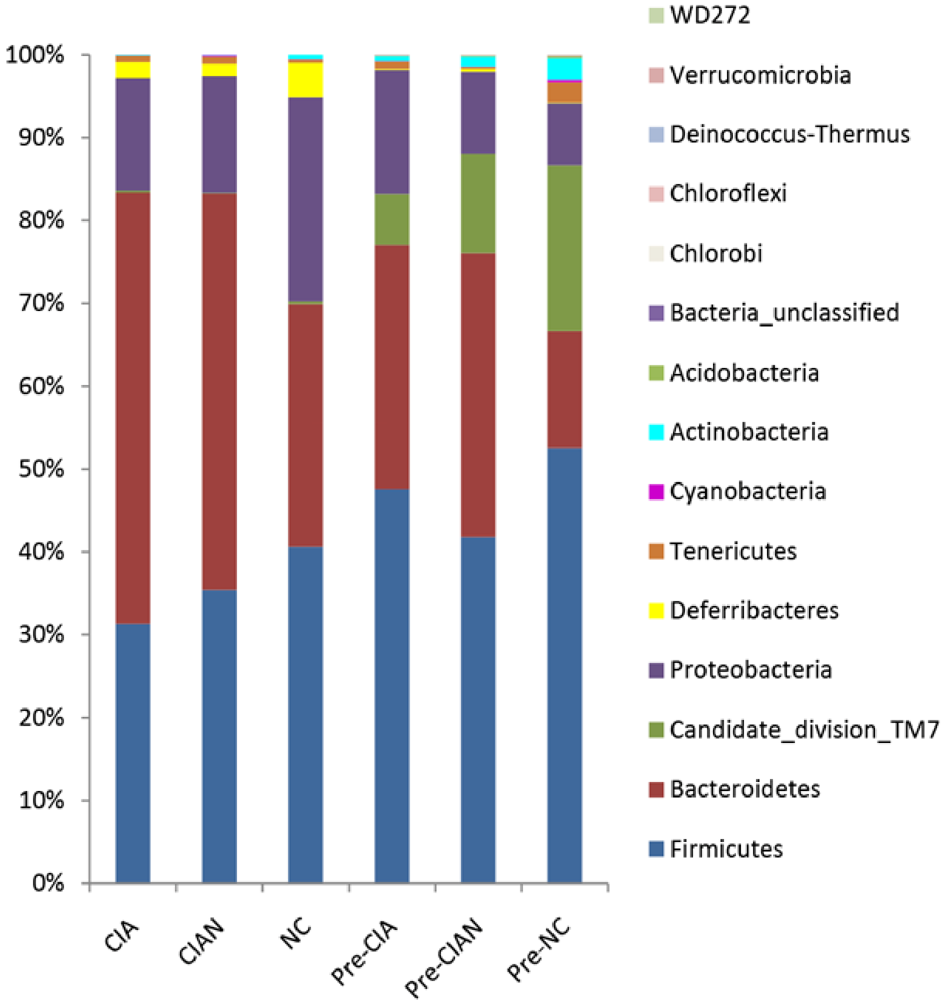

Phyla are plotted according to their relative abundance within each microbiota community, as determined by the number of reads assigned to each phylum. Bars represent the relative abundances of phyla in individual groups.

Supplementary Figure 2. Non-metric multidimensional scaling of OTUs.

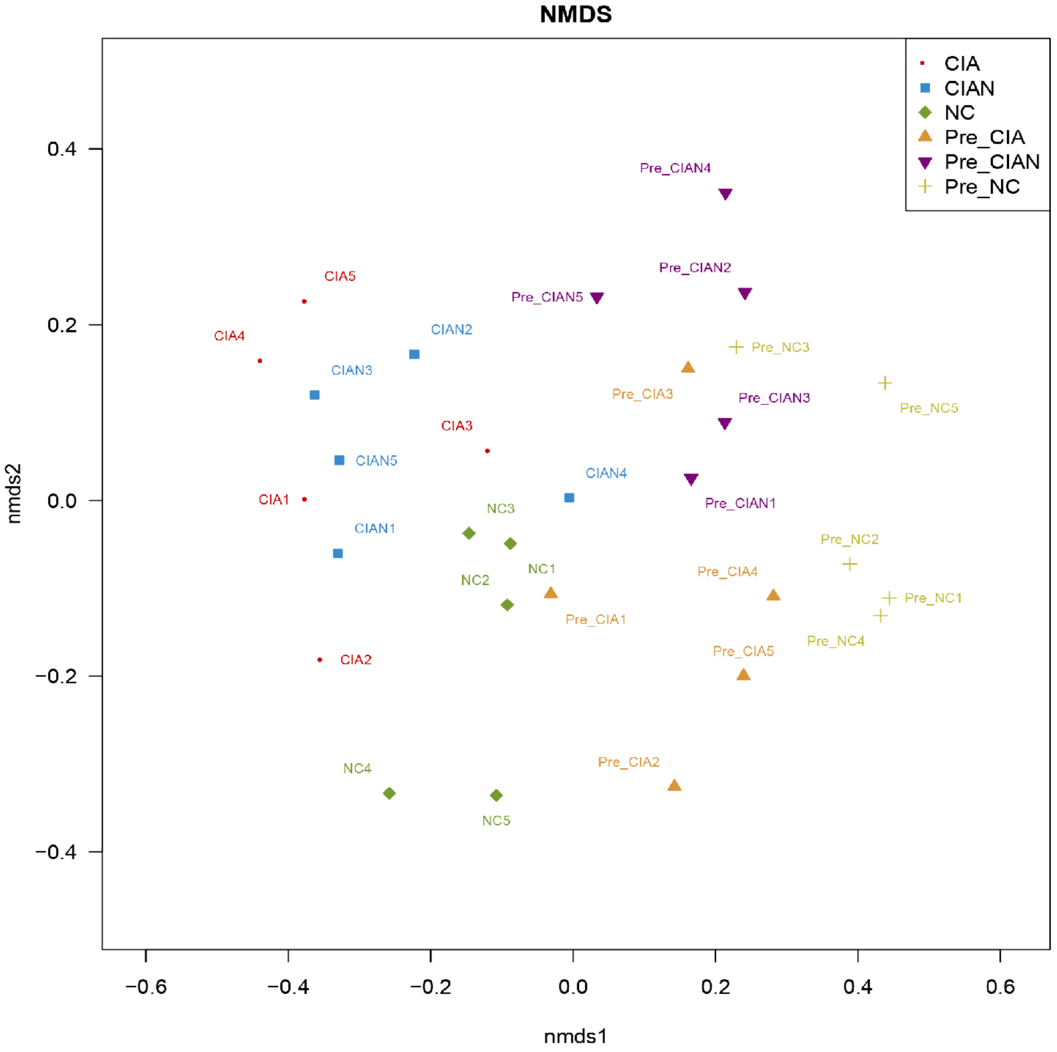

Overall, OTUs showed ≥ 97% identity atthe species level scored using Bray–Curtis similarity analysis. Samples clustered according to CIA-susceptibility and collagen induction. Analysis of similarity (ANOSIM) showed that microbiota composition differed significantly among groups classified according to CIA-susceptibility and collagen induction.

Supplementary Table 1.Analysis of differentially abundant taxa prior to arthritis onset.[a]

Differentially abundant features analysis prior to arthritis onset

Genus	pre-CIA		pre-CIAN		Between-group comparisons p Values
	Mean (%)	s.d	Mean (%)	s.d	Pre-CIA vs Pre-CIAN
**Enterorhabdus**	0.0036	0.0009	0.0107	0.0031	0.0202*
**Alistipes**	0.0919	0.0223	0.1819	0.0364	0.0249*
**Candidatus_Saccharimo**	0.0809	0.0269	0.1770	0.0288	0.0125*
**Anaerotruncus**	0.0023	0.0006	0.0148	0.0040	0.0030**
**Incertae_Sedis**	0.0007	0.0004	0.0044	0.0015	0.0126*
**Intestinimonas**	0.0017	0.0004	0.0057	0.0014	0.0065**
**Lactobacillus**	0.4382	0.0710	0.1907	0.0645	0.0079**
**Desulfovibrio**	0.0309	0.0215	0.1077	0.0266	0.0192*

[a]Symbols indicate significantly different abundances. Abundances with matching symbols were not significantly different from each other. Pre-,prior to arthritis onset; CIA, mice with collagen-induced arthritis; CIAN, mice without arthritis after collagen induction.

Supplementary Table 2.Analysis of differentially abundant taxa after arthritis onset.[a]

Differentially abundant features analysis after arthritis onset.

Genus	CIA		CIAN		NC		Between-group comparisons p Values	
	Mean (%)	s.d	Mean (%)	s.d	Mean (%)	s.d	CIA vs NC	CIA vs CIAN
**Enterorhabdus**	0.0011	0.0003	0.0003	0.0002	0.0014	0.0008	0.0008**	0.0267 *
**Alistipes**	0.0938	0.0090	0.1554	0.0115	0.1170	0.0271	0.5591	0.0008 **
**Desulfovibrio**	0.0257	0.0050	0.0407	0.0212	0.0054	0.0017	0.0017 **	0.5803
**prevotella**	0.0252	0.0056	0.0367	0.0278	0.0012	0.0011	0.0011 **	0.7743
**Myroides**	0.0000	0.0000	0.0001	0.0001	0.0001	0.0000	0.0000 **	0.4240
**Odoribacter**	0.1085	0.0164	0.1130	0.0217	0.0476	0.0106	0.0052 **	0.9150
**Parabacteroides**	0.0077	0.0027	0.0084	0.0040	0.0015	0.0003	0.0238 *	0.9282
**Rikenella**	0.0006	0.0004	0.0003	0.0001	0.0035	0.0014	0.0407 *	0.6695
**Acetatifactor**	0.0008	0.0003	0.0008	0.0005	0.0001	0.0001	0.0259 *	0.9669
**Blautia**	0.0841	0.0227	0.0890	0.0255	0.0288	0.0077	0.0206 *	0.9256
**Brochothrix**	0.0000	0.0000	0.0000	0.0000	0.0002	0.0001	0.0227 *	1.0000
**Coprococcus**	0.0146	0.0063	0.0050	0.0009	0.0017	0.0007	0.0374 *	0.1401
**Incertae_Sedis**	0.0047	0.0016	0.0051	0.0017	0.0011	0.0003	0.0003**	0.6297
**Lactococcus**	0.0009	0.0004	0.0020	0.0009	0.0043	0.0017	0.0415 *	0.2788
**Ruminococcus**	0.0074	0.0032	0.0123	0.0082	0.0008	0.0004	0.0355 *	0.6814
**Streptococcus**	0.0006	0.0001	0.0030	0.0018	0.0053	0.0015	0.0057 **	0.2327
**Pseudomonas**	0.0002	0.0001	0.0013	0.0005	0.0028	0.0014	0.0623	0.0417*

[a]Symbols indicate significantly different abundances (*p<0.05;**p<0.01). Abundances with matching symbols were not significantly different from each other.

CIA, mice with collagen-induced arthritis; CIAN, mice without arthritis after collagen induction; NC, untreated control.
